# Barriers and facilitators of conservative kidney management in older adults with chronic kidney failure: a qualitative study with nephrologists in Germany

**DOI:** 10.1186/s12882-026-05470-3

**Published:** 2026-10-31

**Authors:** Janine Kaiser, Charlotte M. Kugler, Muhammad Helmi Barghouth, Natalie Ebert, Nina Mielke, Maja Krajewska, Elke Schaeffner

**Affiliations:** 1 Institute of Public Health, Charité – Universitätsmedizin Berlin, corporate member of Freie Universität Berlin, Humboldt Universität zu Berlin, Charitéplatz 1, 10117 Berlin, Germany; 2 Institute for Health Services and Health System Research, Faculty of Health Sciences Brandenburg, Brandenburg Medical School (Theodor Fontane), Immanuel Klinik Rüdersdorf, Seebad 82/83, 15562 Rüdersdorf, Germany; 3 Center for Health Services Research, Brandenburg Medical School (Theodor Fontane), Rüdersdorf, Germany; 4 Institute of Biometry and Clinical Epidemiology, Charité – Universitätsmedizin Berlin, corporate member of Freie Universität Berlin, Humboldt Universität zu Berlin, Charitéplatz 1, 10117 Berlin, Germany

**Keywords:** Conservative kidney management, Shared-decision-making, Chronic kidney disease, Older adults, Nephrologists, Relatives, Semi-structured interviews

## Abstract

**Background:**

Conservative kidney management (CKM) offers a treatment option for older adults with chronic kidney failure (CKF) and limited life expectancy. International evidence suggests that nephrologists face challenges in providing CKM. However, in Germany, in-depth research on this topic remains limited. Therefore, this study aims to explore barriers and facilitators experienced by nephrologists in CKM delivery to derive measures for improving its implementation.

**Methods:**

Semi-structured interviews were conducted with nephrologists working in inpatient or outpatient settings in Berlin and the Federal State of Brandenburg, Germany in 2024. The Theoretical Domains Framework (TDF) guided data collection. The data were analyzed using qualitative content analysis.

**Results:**

Eleven interviews were conducted. Nephrologists reported barriers and facilitators across ten TDF domains, overall. Barriers in delivering CKM were identified across nine TDF domains, such as *knowledge* (lack of CKM education), *skills* (insufficient shared-decision-making, SDM), *social/professional role and identity* (lack of clarification of nephrologists’ role in palliative care, divergence from professional role perception), *beliefs about consequences* (caregiver burden), *reinforcement* (unattractive reimbursement), *environmental context and resources* (insufficient intersectoral and interdisciplinary collaboration), and *social influences (*relatives, societal expectations). Facilitators were also reported in nine TDF domains, for example *knowledge* (CKM training), *reinforcement* (adequate reimbursement), *intentions* (adjustment of treatment goals for older patients), *environmental context and resources* (improved interdisciplinary and intersectoral collaboration, patient decision aids), and *social influences* (treatment discussions with and without relatives).

**Conclusions:**

CKM delivery is challenging at multiple levels. Our findings align with international barriers and complement previous national evidence by providing deeper insight into nephrologists’ attitudes, barriers within SDM, and the role of relatives. Improving CKM implementation requires multifaceted strategies, particularly system-level measures such as adequate reimbursement and CKM-related training to strengthen awareness and acceptance. In addition, structured SDM approaches and guidance on involving and considering relatives may be crucial to support CKM as a valid treatment option.

**Supplementary Information:**

The online version contains supplementary material available at https://doi.org/10.1186/s12882-026-05470-3.

## Introduction

With demographic change, the number of older adults is increasing worldwide, alongside chronic disease and chronic kidney failure (CKF) [1]. Worldwide, most patients starting dialysis are at least 75 years old [2]. In Germany, 60% of dialysis patients are aged 65 or older, with highest prevalence among those aged 75 to 84 years [3, 4]. By 2040, the number of nursing home residents undergoing dialysis could rise by 44% [4]. Although kidney-protective therapies such as sodium glucose linked transporter 2 (SGLT2) inhibitors may slow progression, older adults are still expected to remain the largest group affected by CKF [5].

Hemodialysis (HD) is the most common treatment in CKF and is particularly prominent in Germany [6–8]. However, data show that HD is often associated with high early mortality, especially in individuals aged ≥ 75 years with multimorbidity or frailty [9–15]. Conservative care management (CKM) is an active non-dialytic treatment approach focusing on symptom management and maintaining quality of life [16]. Recent data suggest that, compared with CKM, dialysis in older adults may not always provide a clear survival advantage and may reduce time spent at home [17]. However, comparative survival data in older patients remain limited. Given that treatment goals can change with age and many older patients with CKF and limited life expectancy may prioritize quality of life over life prolongation, the choice between dialysis and CKM requires careful consideration [18, 19].

In a European survey, nephrologists estimated that CKM was offered to 10% of patients for whom renal replacement therapy would otherwise have been considered [20]. In Germany, a survey among nephrologists suggested that about 5% of dialysis-eligible patients are treated conservatively [21]. In both studies, CKM referred to a decision not to initiate dialysis rather than to a temporary delay of dialysis initiation. However, these data should be interpreted cautiously, as they were based on nephrologists’ self-reported estimates and the German survey was limited to one non-profit provider type. International guidelines encourage outpatient nephrologists to initiate early education on available treatment options once patients are at risk for CKF, as part of broader advance care planning [16]. German data suggest that older patients may experience insufficient shared decision-making (SDM) and feel inadequately informed about alternatives to HD, which may indicate that HD is often perceived by nephrologists as the preferred treatment option [22, 23].

International studies have identified structural and organizational and decision-making barriers to CKM implementation, including lack of nephrologist training, higher financial incentives for dialysis, absent CKM-specific guidelines, high time demands, poor interdisciplinary collaboration and difficulties in estimating prognosis or discussing death, and dealing with emotional aspects [24–27]. However, these barriers are likely influenced by a range of contextual factors, such as the health system, organizational structures, and cultural aspects, and may therefore vary between countries.

In Germany, a survey among non-profit dialysis centers identified similar barriers to CKM delivery, such as limited palliative care structures, staff shortages, limited resources, and legal uncertainty [21, 28]. However, the survey mainly captured structural barriers providing limited insights into how nephrologists perceive CKM or discuss it with patients. A deeper and more comprehensive understanding of the challenges nephrologists face in offering CKM is therefore needed.

### Objective

This study therefore aims to answer the following research question: What barriers and facilitators do nephrologists in Germany experience in CKM delivery? Based on these findings, targeted measures to promote CKM implementation will be derived.

## Methods

Semi-structured face-to-face interviews were conducted one-on-one by JK with nephrologists at their workplaces. The study follows the consolidated criteria for reporting qualitative research (COREQ) [29]. The research team included health services researchers and nephrologists. Team characteristics and reflexivity are described in Additional file 1.

### Sampling and recruitment

To capture a heterogeneous sample, maximum variation sampling was applied to identify a broad range of barriers and facilitators. A sampling plan considered regional variation and organizational diversity. Following Küpper’s typology, participants were recruited from urban Berlin and rural Brandenburg [30]. Recruitment aimed to include nephrologists from non-profit, industrial, and private dialysis providers, independent practices, and outpatient clinics. The sampling plan was iteratively revised during data collection, leading to inclusion of inpatient nephrologists.

Recruitment took place between July and October 2024 via telephone contact with existing contacts of the study team, as well as email and postal invitations. Snowball sampling was additionally applied. Recruitment concluded once data saturation was reached, defined as the point at which at least two consecutive interviews did not generate new codes or subcodes [31].

### Data collection

The interview guide (Additional file 2) was developed following Helfferich, based on previous research and guided by the Theoretical Domains Framework (TDF) [32, 33]. The TDF is a validated implementation framework, synthesizing 33 behavior change theories and 128 constructs into 14 domains to systematically identify barriers and facilitators in healthcare [34, 35]. Its application followed established guidelines [35]. The guide was revised after feedback from experienced researchers and subject-matter experts (ES, NE, CMK, NM) and covered four key topics (Table 1). It was pilot tested for clarity with one research team member (NE) and one person outside the study team who was not a nephrologist, and was adapted accordingly. Interviews were audio-recorded using the PHILIPS Digital Voice Tracer 1120 (Hamburg, Germany).

**Table 1:** Interview guide topics and key questions

Topic	Key question
Decision-making process	How do you perceive the decision-making process regarding treatment options?
Delivery of CKM	How feasible is it for you to follow the pathway of CKM in your daily routine?
Perception of CKM	What beliefs or opinions do you have about CKM?
Barriers and facilitators in implementing CKM	What do you think are the barriers and facilitators to implementing CKM (in your daily routine)?

CKM - Conservative Kidney Management

### Data processing and analysis

Interviews were transcribed verbatim using NoScribe (Version 0.5, 2023), checked for accuracy, and pseudonymized by JK. Data were analyzed using Kuckartz’s qualitative content analysis, combining deductive TDF-based categories with inductive categories [36]. Coding tree development was conducted using MAXQDA (Version 24.0, VERBI Software, 2023). JK and MHB independently coded the first three transcripts and discussed discrepancies until consensus was reached. The revised coding framework was then applied by JK to the remaining transcripts and further refined throughout the analysis, with any uncertainties being addressed through discussions with the research team. Revisions were subsequently applied to previously coded material where necessary. Data saturation was assessed iteratively during ongoing data collection and analysis by monitoring in MAXQDA whether new main codes or subcodes emerged across consecutive interviews. Quotes were translated into English and then back-translated using ChatGPT (v5.5, OpenAI, San Francisco, USA, 2026). All quotes were fully anonymized prior to translation, ensuring that no personal or identifiable information was entered into ChatGPT. To verify translation accuracy, the English translations were additionally reviewed against the original German quotes by a bilingual person and revised.

## Results

Eleven interviews were conducted by JK between August and October 2024. Due to failed recording, one interview was repeated. Interviews lasted 19–55 min (mean = 29 min). Data saturation was reached after the 11th interview, as no new code or subcode emerged from the data within the last two interviews. The sampling plan was adapted during data collection due to difficulties in recruiting nephrologists, especially those from commercial kidney and dialysis centers and rural areas. The initial three interviews revealed relevance of CKM also for the inpatient setting, leading to inclusion of inpatient nephrologists. A final sample is described in Table 2.

**Table 2:** Sample and characteristics

Characteristics	Number of inteviewees	
**Institution**	urban	rural
Outpatient non-profit nephrology and dialysis center	0	1
Outpatient industrial nephrology and dialysis center	0	0
Outpatient private nephrology and dialysis center	3	1
Outpatient practice (no affiliation to dialysis services)	1	0
Outpatient nephrology service in a hospital	2	0
Inpatient nephrology department in a hospital	3	0
**Sex**		
Women	7	
Men	4	
**Clinical experience in nephrology**		
≤ 20 years	4	
> 20 years	7	

The majority of nephrologists (82%, *n* = 9) worked in an urban area, and 64% (*n* =7) were women. On average, participants had 25 years of experience in nephrology (range: 10–45 years). The sample was heterogeneous regarding workplace setting, including inpatient and outpatient care as well as different organizational structures.

### Barriers and facilitators for implementing conservative kidney management

Barriers and facilitators were analyzed separately, each mapping to nine TDF domains. Overall, 10 different TDF domains were represented. Barriers and facilitators are presented jointly within each main category where applicable and supported by illustrative participant quotes. The results are reported following the domain order of the TDF. An overview of all barriers and facilitators is provided in Table 3.

**Table 3:** Barriers and facilitators in providing conservative kidney management

TDF domain / main category	Barrier	Facilitator
*Knowledge*	Lack of knowledge *No training in CKM* *Biased information* *No training in SDM* *Withholding information from patients*	Training of nephrologists in CKM Training of nursing staff in CKM Research on CKM implementation Experience
*Skills*	Insufficient SDM *Unstructured decision-making-process* Uncertainty about conducting CKM	Empathetic and unbiased patient education
*Social/professional role and identity*	Divergence from professional role CKM as a passive treatment option Withholding of treatment Lack of clarification of nephrologists’ role in palliative care Lack of acceptance of CKM Lack of acceptance of patient preferences Assumptions about patient preferences	Exploring patients’ wishes and needs
*Beliefs about capabilities*		Confidence in CKM delivery
*Beliefs about consequences*	Lower quality of care for patients with poor adherence Lack of social interaction for patients Caregiver burden Legal consequences	
*Reinforcement*	Unattractive reimbursement Missing recognition of end-of-life support	Adequate reimbursement
*Intentions*	Preserving life	Adjustment of treatment goals for older patients Ethical practice
*Memory*,* attention and decision processes*	Uncertainty in predicting treatment trajectories	Reassessment of treatment
*Environmental context and resources*	Insufficient interdisciplinary collaboration *Lack of communication between disciplines* *Unclear resposibilities between disciplines* *Inadequate consideration of nephrology patients in palliative care* *Lack of integration of palliative care in nephrology* Lack of intersectoral communication *Difficult transition to outpatient nephrological care* No capacity for home visits Transportation barriers Missing CKM-specific guideline and standard operating procedures Personnel and time requirements Time pressure in critical situations	Improved interdisciplinary and intersectoral collaboration *CKF patient card* Early patient education on treatment options Patient decision aids Specialized CKF treatment center
*Social influences*	Wishes of patients and relatives Presence of relatives in treatment discussions Death as taboo topic Societal expectation of medical treatment in older adults with limited life expectancy to prolong survival time	Treatment discussions without relatives Treatment discussions with relatives

TDF - Theoretical Domains Framework; CKM - Conservative Kidney Management; SDM – Shared-Decision Making; Terms in italics represent sub (sub)categories within the corresponding subcategories

#### Knowledge

In the *knowledge* domain, nephrologists reported limited knowledge of SDM and CKM-related topics, involving palliative care and end-of-life discussions, as these topics are not covered in medical school or board certification nephrology training. Limited knowledge of CKM was reported to result in CKM being either not discussed or presented in a biased way, for example as a choice between dialysis and death.

*There are many physicians who the patient*,* well*,* if you don’t want dialysis*,* then do you want to die? And then the patient is already so shocked and says*,* well (…) I don’t want to die that soon. And then the physician says*,* well*,* then you have to choose dialysis. And the fact that with conservative treatment you don’t die immediately (…) and may perhaps have a better time than with dialysis*,* is not discussed at all*,* because I think many physicians simply don’t know this*,* how to actually manage it [CKM].* (Interview 2, outpatient sector)

Participants emphasized CKM training curricula, training for dialysis nurses to support home-based CKM and implementation research. Awareness of and confidence in CKM were reported to develop mainly through clinical experience.

With increasing experience, nephrologists reported being more able to accept the possibility of earlier death in older patients undergoing CKM, as they become more aware of disadvantages of dialysis in this age group.

*I think one can more easily accept the possibility of an earlier death in a patient when one has seen many different dialysis courses*,* simply because with age and experience*,* one has witnessed more. At the beginning*,* one is certainly more focused on trying to save everyone.* (Interview 7, outpatient sector)

#### Skills

Within the Skills domain, interviewees reported that some nephrologists lack the communication skills needed to apply SDM in practice. In particular, some nephrologists were reported to lack structured communication strategies for guiding patients through the decision-making process. Interviewees described approaches ranging from paternalistic decision-making to asking patients for their treatment preference without first providing sufficient information on treatment options.

*Communication skills are simply not taught. I only learned them when I was already a specialist and worked on a palliative care ward. Specialist training does not teach nephrologists how to communicate well by bringing together the medical indication*,* the prognosis*,* and what the patient wants. There’s one way: I state the medical indication*,* I tell that to the patient*,* and then I walk out the door. And then there’s the other way*,* which I think is just as stupid. I ask the patient what they want. That’s totally ridiculous*,* and that’s actually what happens these days. What would you like? And then they all sit there thinking*,* I have no idea what’s good for me. And then they think they have to answer*,* because we asked them*,* so they just say something.* (Interview 6, outpatient sector)

Thus, nephrologists pointed out the importance of empathetic and unbiased communication to avoid creating emotional pressure that might contribute to patients excluding CKM as a treatment option. Additionally, uncertainty in actively applying CKM was reported, particularly in managing symptoms such as dyspnea, due to limited experience in palliative nephrology.

*And well*,* the fear of suffocating and shortness of breath is probably quite an issue. Which would then require the use of opioids. And to just give that en passant*,* I would also find difficult as a doctor not familiar with palliative medicine.* (Interview 5, outpatient sector)

#### Social/professional role and identity

With regard to their social and professional role and identity, nephrologists described CKM as conflicting with their self-perception as active practitioners. CKM was often perceived as doing nothing rather than as an active treatment option, leading to limited discussion of CKMand concerns about withholding treatment from patients.

*So to speak*,* folding your hands in your lap and doing nothing when a patient is dying or is feeling bad*,* that’s simply not a doctor’s understanding of their role.* (Interview 9, inpatient sector)

In this context, interviewees questioned CKM and palliative care as a treatment option in their responsibility.

*It is the decision about whether palliative nephrology should actually be part of the nephrological canon. One can say*,* I limit myself to the active form. If the patient*,* so to speak*,* needs conservative kidney management*,* and also considering that the nephrologist is not really trained for that*,* then I pass on that responsibility. That*,* so to speak*,* is not my playing field.* (Interview 4, outpatient sector)

According to interviewed nephrologists, physicians often assume they know what is best for the patient, frequently presuming that survival is always the patient’s primary goal. Even when patient preferences are known, they are not always fully respected, and patients may still be persuaded to start dialysis against their wishes.

*We recently had a patient who actually died during her first dialysis session. She always said she didn’t want dialysis*,* but was persuaded to have a shunt placed. Every time I saw her*,* she would say*,* not yet*,* and so on. And then she came in and said*,* today I’m going to die*,* and that’s exactly what happened. It really makes you wonder whether it was right for us as physicians to convince her.* (Interview 5, outpatient sector)

Some nephrologists emphasized the need to actively explore and follow patients’ wishes during decision-making. This was considered essential to achieve patient-centered decisions that lead to greater satisfaction for both patient and physician.

#### Beliefs about capabilities

Nephrologists underscored that positive feedback from patients’ relatives give them confidence in discussing and implementing CKM. This confidence reinforced their view of CKM as an important treatment option.

*I think this positive feedback [from relatives] has also strengthened me*,* and therefore I feel comfortable in these discussions. (…) I have been practicing it [CKM] for a long time now*,* and it gives me satisfaction. When I see that patients are happy because their wishes are respected*,* then I know CKM is a good concept.* (Interview 2, outpatient sector)

#### Beliefs about consequences

The interviews revealed several *beliefs about consequences* of CKM. Dialysis was considered preferable some older patients living alone because regular sessions provide medical care and social interaction, whereas CKM was assumed to involve fewer visits and less contact, reducing mental well-being. Dialysis was also perceived to relieve relatives through regular professional contact and time for daily tasks, given reassurance that their loved ones were cared for. Further concerns included legal uncertainty and poorer CKM outcomes in patients with low adherence, for whom dialysis may provide a more structured care setting.

*That really is a striking aspect*,* and I think it’s one that shouldn’t be overlooked. The fact that someone checks on them three times a week and basically cleans everything out again. For some*,* it’s really the only chance to get better*,* because they don’t watch their fluid intake*,* they can’t get their diabetes under control*,* they don’t take their blood pressure medication*,* and so on. And I’ve already had the feeling sometimes that they’ll just be placed into a care system.* (Interview 6, inpatient sector)

#### Reinforcement

Regarding the *reinforcement* domain end-of-life care was perceived as less valued than technical interventions, resulting in limited recognition for providing CKM. Low reimbursement was identified as a major barrier, as fixed per-patient payments in outpatient nephrology do not account for the time-intensive communication and intensive care often required in CKM. Adequate reimbursement was highlighted as key for CKM implementation and improved patient access.

*That means the doctor doesn’t get any additional reimbursement*,* that means we basically get a certain amount of money per case each quarter. But if we then say that the patient receiving conservative treatment actually needs to be seen every two weeks*,* then that’s something the doctor has to cover personally*,* and it has to be cross-financed from the remaining profits the practice generates.* (Interview 2, outpatient sector)

#### Intentions

Within the *intentions* domain, some nephrologists indicated that the primary intention of nephrologists is often to preserve life, which is why CKM is rarely considered as a treatment option.

*And I think it [end of life] is something we don’t like to talk about*,* because we’re trying to keep people alive.* (Interview 5, outpatient sector)

In this context, interviewees voiced the need to differentiate treatment goals for older patients with limited life expectancy and emphasized that maintaining quality of life should be considered a primary goal for this group.

*We need to make distinctions*,* as we are dealing with different patient groups. Conservative kidney management may in some ways conflict with our initial assumptions*,* but we have to recognize these differences. We would not refer every patient for transplantation*,* and therefore we need to learn what the treatment goal should be at an advanced age. In my view*,* that goal is primarily quality of life.* (Interview 1, inpatient sector)

Ethical responsibility was described as an important facilitator of CKM, emphasizing that patients should receive the most appropriate treatment regardless of financial barriers.

#### Memory, attention and decision processes

Within the *memory*,* attention and decision processes* domain, nephrologists reported difficulties estimating patients’ survival due to clinical complexity, such as multimorbidity, often leading to avoidance of prognosis discussions.

*I’ve never told a patient how many months or days they have left to live. I would find it very difficult to do that.* (Interview 5, outpatient sector)

Some nephrologists mentioned the importance of reassessing treatment decisions to ensure the care remains aligned with patients’ changing needs.

#### Environmental context and resources

Within the *environmental context and resources* domain, nephrologists criticized unclear roles and responsibilities in interdisciplinary care, with poor communication often leading to conflicting decisions and misunderstandings of patient needs.

*(…) I also don’t understand it when a patient is seeing a cardiologist*,* has an extremely limited prognosis*,* and the cardiologist gets upset that I’ve stopped the statin because the patient has triple-vessel coronary disease. (…) According to the official interpretation*,* it reduces the (…) ten-year risk of having a secondary event. Ten-year risk. Are we talking about the next few weeks here*,* or about ten years? And a statin can also cause side effects*,* after all.* (Interview 11, outpatient sector)

In terms of interdisciplinary care, nephrology patients were reported to have limited access to palliative care, as services are often prioritized for patients with oncological or other terminal illnesses. The lack of integrated palliative care in nephrology was reported to contribute to potentially avoidable hospital admissions during clinical deterioration, as general practitioners may refer patients to inpatient care when symptom management becomes challenging.

Both outpatient and inpatient nephrologists identified deficits in intersectoral communication. The transition from inpatient to outpatient care was described as challenging for CKM patients, as outpatient nephrologists are often at capacity due to dialysis care, making timely follow-up difficult.

*So*,* dialysis patients are seen by a doctor three times a week in the outpatient setting. That also means*,* for example*,* if it’s all justifiable*,* we tend to discharge dialysis patients more quickly. Because we know tomorrow*,* they’ll be back in their outpatient clinic*,* they’ll have another doctor’s visit*,* so nothing critical will be missed. And it [CKM] is exactly the opposite with how things are at the moment. If we discharge a patient now*,* in some cases we can’t even get a proper follow-up appointment in the outpatient clinics on short notice. So*,* we sometimes have major problems getting appointments in the outpatient setting for non-dialysis patients. *(Interview 8, inpatient sector)

Improved interdisciplinary and cross-sectoral collaboration was emphasized to prevent misunderstandings and unnecessary hospitalizations. A CKF-specific patient ID card was suggested to facilitate communication of treatment decisions in emergencies.

*(…) for various diseases*,* there are small identification cards that patients carry with them. There’s also the emergency card that lists the blood type and maybe allergies and so on. So*,* if*,* for example*,* it says that the patient has chronic kidney disease stage 4 or 5 and is treated conservatively that could of course help*,* because many patients may not be able to explain that very clearly themselves.* (Interview 3, outpatient sector)

Interviewees noted that transportation costs for patients receiving CKM are frequently not covered by health insurers due to increased administrative requirements, unlike for dialysis patients.

*In the end*,* for someone who is receiving outpatient care and conservative therapy*,* there is certainly the option of arranging medical transport to bring the patients in and then take them back home afterwards. But the hurdles are quite different. You definitely need to have a severe disability*,* and most of the time you also need to have a care level. And those aren’t things you can just get ad hoc. So*,* in the end*,* the patient has to make an effort themselves.* (Interview 10, outpatient sector)

In addition, the lack of capacity for home visits, which would be indicated given the frequent physical impairments in this patient group, was highlighted.

Nephrologists also criticized the absence of dedicated CKM guidelines and standardized operating procedures leaving care dependent on individual physician experience and preferences. When patients first present with CKF, urgency may lead to dialysis by default, leaving little time for patient-centered decision-making. Further time constraints were highlighted, as CKM requires more time and personnel resources than dialysis, particularly for comprehensive discussions and care delivery.

*Both the information consultation and conservative kidney management itself are very time-consuming. Especially information consultation (…) requires discussing the alternative in a way that is understandable*,* which will probably take several appointments. And (…) the initiation of dialysis treatment is something that can be done more quickly and with less effort*. (Interview 1, inpatient sector)

Interviewees emphasized early, comprehensive education on treatment options to ensure that CKM is considered and informed decisions are possible. A structured patient decision aid (PDA) was suggested to support SDM, particularly for less experienced nephrologists, by incorporating frailty, comorbidities, and patient preferences, and by guiding conflicts between medical recommendations and patient wishes. Dedicated CKM clinics were also proposed to improve care continuity and inpatient-outpatient transitions.

#### Social influences

Within the *social influences* domain, nephrologists reported that patient and family preferences for dialysis were often followed, even when dialysis was not recommended. Relatives’ involvement was perceived as ambivalent, as their presence may hinder end-of-life discussions but also help align decisions with patient preferences and prevent misunderstandings or conflicts. Selective involvement of relatives was identified as a facilitator, allowing patients to express preferences independently while still receiving family support when appropriate.

*Maybe these conversations should also be conducted alone with the patients*,* because these conversations are often held with family members*,* and they actually do not want to hear about the topic [end of life]. (Interview 5*,* outpatient sector)*

Participants also described a societal expectation that all available medical interventions should be used to prolong life, regardless of patient age or clinical prognosis.

*And there is this kind of mindset in Germany that people consume a lot. That older patients*,* even 90-year-olds with a tumor*,* still receive cancer treatment. So there really is this sense of entitlement as I want everything that is somehow possible.* (Interview 1, inpatient sector)

## Discussion

This study explored nephrologists’ perspectives on CKM implementation in Germany. Our findings suggest that interacting multilevel factors influence nephrologists’ behavior and may hinder CKM delivery.

Insufficient CKM-related education was identified as a central system-level barrier, consistent with US and Canadian data showing that limited training may reduce CKM discussions [24–26, 37]. US training programs such as NephroTalk, can improve nephrology trainees’ knowledge and self-reported preparedness for CKM and related communication skills, supporting integration of CKM training into nephrology education [38]. In Germany, a structured curriculum for palliative nephrology including CKM has been proposed [39]. However, to our knowledge, no implementation program for this curriculum has been developed. Broad implementation in medical education and nephrology training is needed to strengthen nephrologists’ role in CKM.

Financial incentives were identified as another major system-level barrier. Although reimbursement systems differ internationally, evidence suggests existing structures often favor dialysis-centered care [27]. In Germany, outpatient nephrologists receive a fixed quarterly payment per CKM-patient, regardless of care needs, creating potential disincentives given the time-intensive communication and support required. From a health system perspective, CKM may be less resource-intensive than dialysis in the long term and may reduce overall costs, suggesting a mismatch between potential system-level savings and provider-level incentives [4]. Similar concerns were reported in the US, where nephrologists described strong financial incentives to dialyze patients and insufficient reimbursement for in-depth CKM discussions [24–26]. In UK, separate funding mechanisms, with dialysis funded centrally and non-dialytic management locally, were described as potentially limiting resources for CKM programs, particularly in rural areas [24]. Within the broader context of financial incentives, provider type may also play a role. European survey data suggest that, compared with those in for-profit centers, nephrologists in not-for-profit centers were more likely to consider patient preferences important when deciding on CKM and to discuss decisions to forgo renal replacement therapy with patients and families [20]. Recent US commentary also raises concerns that large for-profit dialysis organizations may reinforce standardized, dialysis-centered care and limit flexibility for individualized care and innovation [40].

In line with a previous study from Germany, nephrologists in our study emphasized the need for reimbursement structures that better reflect the actual workload in CKM [28]. In Germany, a systematic assessment of CKM-related time and personnel requirements may be an initial step to inform reimbursement adjustments to the outpatient Uniform Value Scale (Einheitlicher Bewertungsmaßstab), regulating reimbursement for outpatient medical services.

SDM emerged as a key area in which barriers may arise before CKM is delivered. Treatment options were said to be presented unstructured or biased, including framing CKM as a choice against life-prolonging treatment. Previous German studies suggest that patients often feel insufficiently informed, that CKM is rarely considered, and nephrologists are uncertain in end-of-life discussions [21–23, 28]. Our findings extend this evidence by showing how communication practices may further limit informed, preference-sensitive decision-making and contribute to inappropriate care [41]. This aligns with US and UK evidence showing that patients and families may perceive the decision as one between dialysis and death, while patient information materials often frame dialysis as treatment and CKM as non-treatment [42–44]. Our findings therefore highlight the need for CKM-related communication training and structured SDM training. Although an SDM-focused curriculum has been introduced in Germany medical education, many practicing nephrologists trained before its implementation [20, 40]. Within the German context, programs such as Share-to-Care have improved SDM in hospital settings, including nephrology, but have not yet been evaluated in outpatient nephrology practices [45]. A targeted US educational intervention for outpatient nephrologists suggests improvement in clinician confidence in SDM for older patients, including CKM [46].

Discussing prognosis represents a key element of SDM [2]. However, nephrologists often avoid these discussions due to clinical complexity. Most older patients prefer to receive prognostic information, even if uncertain [47]. Without it, patients may underestimate disease severity and not consider CKM [48]. Nephrologists suggested the use of a PDA to facilitate SDM. Evidence suggests that PDAs can support SDM in advanced chronic kidney disease for both patients and clinicians [49, 50]. Although a German PDA supporting the decision between renal replacement therapy and CKM is available, it was developed within a SDM program for the hospital setting and was not specifically developed for older patients as the primary target population or to provide specific prognostic information for this population [45, 51]. Given that prognosis is a crucial element of SDM, development of a PDA, informed by prognostic models for older adults, may be a key advancement in promoting SDM. Although prognostic models exist, their performance and clinical usability remain limited [52]. To address this barrier, a PDA supporting decision-making between dialysis therapy and CKM for older adults, informed by a prognostic model, is currently being developed in Germany as part of the CHOICE project, funded by the Innovation Committee of the Federal Joint Committee (funding code: 01VSF24025).

Nephrologists also reported that CKM may conflict with their professional self-understanding as active, life-prolonging practitioners and may be perceived as withholding rather than providing treatment, consistent with findings from the US, UK, and Canada [27, 37]. However, this perception conflicts with the current understanding of CKM as active care involving components, such as symptom management, advance care planning, psychosocial support, and ongoing reassessment [2, 53]. It may also reflect an overestimation of the survival benefit of dialysis in older adults, as recent evidence suggests that dialysis in older adults not always associated with a meaningful survival advantage and may reduce time spent at home [17]. Integrating CKM into nephrology training and developing dedicated clinical guidelines is needed to strengthen knowledge, clarify nephrologists’ role and reframe CKM as active care.

Nephrologists in this study described a taboo surrounding death, which may make CKM discussions uncomfortable and contribute to incomplete patient information, aligning with findings from the US and UK [24, 26]. Such discomfort may reflect limited societal acceptance of treatment approaches that do not necessarily aim to prolong life, a barrier also described by participants in this study, thereby reinforcing dialysis as the default option. A similar tendency toward maximal treatment despite poor prognosis has been described in German intensive care, where high use of mechanical ventilation was observed in older patients and was associated with high in-hospital mortality [54]. In this context, the broad availability of resources and high reimbursement have been discussed as factors that may encourage potentially non-beneficial treatment, suggesting that limited financial or structural constraints can reduce the need to address treatment limitation and end-of-life discussions. Similar mechanisms may be relevant in dialysis care and may reinforce both dialysis as the default option and societal expectations of maximum life-prolonging treatment. Providing patient education materials that present balanced information on survival outcomes, treatment burden, and quality-of-life implications may help increase public awareness of CKM and support understanding that good end-of-life care should reflect individual priorities rather than life prolongation alone.

Relatives’ involvement in SDM was perceived as ambivalent. While it may hinder open end-of-life discussions, it can also support alignment with patient preferences. Accordingly, nephrologists suggested that relatives should not be present in all treatment discussions to facilitate open communication. Previous research from Germany show that relatives are involved in decision-making and may experience conflict when dialysis is withheld [21]. Our findings complement these findings by showing how their involvement influence the decision-making process for nephrologists. Guidance on how to involve relatives remains sparse. Given the close interdependence between patients and relatives in this population, a family-centered approach to SDM may be beneficial while still ensuring patient autonomy [55].

Nephrologists reported concerns about increased caregiver burden, assuming dialysis provides greater psychosocial support. While treatment decisions should be guided by medical indication and patient preferences, caregiver burden may influence treatment decision and should therefore be considered [55–57]. Evidence suggests that caregiver burden is comparable between CKM and dialysis, although their needs often remain insufficiently addressed [56, 58]. While international evidence indicates that supportive and peer-support interventions can reduce caregiver burden in relatives of CKF-patients, evidence on how caregivers are considered and supported in Germany remains scarce, highlighting the need for further research on this topic to ensure adequate support and alignment with patient preferences in SDM [59]. To our knowledge this barrier has not been reported in previous international studies in this context.

Despite growing international recognition of CKM as active care for patients with CKF who may not benefit from dialysis, implementation remains challenging [20, 27]. Our findings align with international system-level and organizational barriers, including financial disincentives, insufficient CKM and communication education, absence of dedicated guidelines, limited interdisciplinary and intersectoral collaboration, and lack of implementation research [27, 28, 60]. As these barriers reflect healthcare structures and influence nephrologists’ motivation, they may be considered as key drivers of CKM implementation. The International Society of Nephrology has proposed an action plan addressing these drivers [60]. However, implementation cannot follow a one-size-fits-all approach. These strategies must be adapted to each national healthcare context. In Germany, efforts should initially focus on system-level and organizational barriers, particularly CKM education, reimbursement, guidelines, and coordinated care pathways. Addressing these barriers may also influence other challenges, as improving knowledge is likely to influence attitudes toward CKM. At the same time, SDM should be considered equally important, as it shapes how options are communicated, how relatives are involved, and whether CKM is presented as a valid option in clinical practice.

### Strength and limitations

Variation in professional experience and institutional settings broadened the range of perspectives among nephrologists. Despite the small sample size, data saturation was achieved. However, recruitment was limited to Berlin and Brandenburg, Germany, with no industrial dialysis providers and few rural nephrologists represented. Structural and organizational conditions may differ across nephrology facilities, including the organization of renal palliative care, while specialist palliative care may be more available and accessible in urban than in rural areas [21, 61, 62]. Some perspectives may therefore be missing or underrepresented, limiting the transferability of our findings, particularly to rural nephrology care. Although recruitment sought to capture variation, the study was not designed to provide representative findings for each setting. Individual participants cannot be assumed to represent their provider type, given potential variation of perspectives within settings [21]. Variation in barriers and facilitators across settings was not analyzed, leaving the setting-specific relevance of themes unclear. Most participants had extensive clinical experience, which might have increased their exposure to and awareness of CKM. Selection bias cannot be excluded, as nephrologists supporting CKM may have been more likely to participate, potentially overrepresenting awareness and acceptance while underrepresenting critical perspectives.

Barriers were not prioritized by participants, limiting conclusions about their relative importance. Interpretation and proposed measures focused on frequently reported or particularly important barriers and new additional insights specific to German healthcare. The measures are therefore not exhaustive. Further, the shorter duration of some interviews may have resulted in certain perspectives being explored in less detail. However, interview length depended on how much participants had to say about the topics discussed. Shorter interviews still provided focused and in-depth insights, and all interviews covered the topics of the interview guide. To avoid suggestive questioning, open-ended and neutral questions were used. As participants were not explicitly prompted to discuss contradictory views or negative cases, some may not have been captured despite considering differing perspectives in the analysis. Only the first three interviews were independently coded by two researchers, while the remaining interviews were coded by one researcher. Although uncertainties were discussed within the team, this may have increased the risk of interpretive bias. Finally, categorization of barriers within the TDF required some subjective interpretation. When statements could have been assigned to more than one TDF domain, they were assigned to the domain that best matched their meaning and context, which may have influenced the classification of findings.

### Future research

Future qualitative research should examine differences in barriers and facilitators across nephrology care settings and urban and rural contexts, while larger surveys could assess their prevalence and perceived importance to guide prioritization of targeted implementation strategies. As only nephrologists were interviewed, the findings are limited to their perspectives and do not represent the multidisciplinary CKM care pathway as a whole. Future research should therefore incorporate perspectives from other groups, such as general practitioners, geriatricians, palliative care professionals, nurses, psychologists, patients, family caregivers and representatives of health insurance providers. In addition, involvement of health policy makers is crucial to address system-level barriers. Based on these perspectives, measures should be further modified.

## Conclusions

CKM appears less established than dialysis in routine nephrology care. Our findings align with internationally reported barriers to CKM implementation and extend this evidence by highlighting the influence of relatives on CKM decision-making. In the German context, our study further expands previous evidence by providing in-depth insights into nephrologists’ perceptions, communication practices, and system-level challenges. System-level barriers, such as limited CKM-related knowledge and inadequate reimbursement play a central role in its implementation. However, barriers also arise prior to CKM delivery within the SDM process. To establish CKM as a valid treatment option, targeted measures need to be applied.

## Supplementary Information

Below is the link to the electronic supplementary material.

Supplementary Material 1

Supplementary Material 2

## Data Availability

All data supporting the findings of this study are available within the paper.

## Abbreviations

CKM: Conservative Kidney Management
CKF: Chronic Kidney Failure
TDF: Theoretical Domains Framework
SDM: Shared-Decision-Making
HD: Hemodialysis
PDA: Patient Decision Aid
