# Supplementary material for: Barriers and facilitators of conservative kidney management in older adults with chronic kidney failure: a qualitative study with nephrologists in Germany
Source: BMC Nephrol. 2026 Oct 31;27:646. doi: 10.1186/s12882-026-05470-3 (PMC13732868; doi:10.1186/s12882-026-05470-3)
Supplement: Supplementary file 1 [file 12882_2026_5470_MOESM1_ESM.docx]

**Additional file 2: Interview guide**

## Research question

## What barriers and facilitating factors exist for nephrologists working in (outpatient) care regarding the implementation of conservative kidney management for older patients with chronic kidney failure?

## Introduction

Thank you very much for your willingness to participate in this study. Before we begin, I would like to provide you with some important information about the study. This study is conducted as part of my master’s thesis as a pilot for a project funded by the Innovation Fund. It focuses on conservative kidney management in older people with chronic kidney failure.

The aim of today’s interview is to learn about your expertise and perspectives regarding conservative kidney management in older patients with limited life expectancy. With your professional support, we hope to gain valuable information about barriers and facilitating factors that are relevant for the systematic and sustainable implementation of this treatment pathway.

[Provide study information, obtain informed consent, start audio recording]

## Opening questions

What do you understand by conservative kidney management in older patients with chronic kidney failure and limited life expectancy?

What role does conservative management play for you as a treatment option in older patients with chronic kidney failure and limited life expectancy?

## Interview guide

| **Key questions** | **Checking aspects** | **Concretising questions** | **Questions for maintenance** |
| --- | --- | --- | --- |
| *Topic 1: Decision-making process* | | | |
| How do you perceive the decision-making process regarding treatment options? | - Providing information about all potential treatment options | To what extent do you inform patients in this patient group about all treatment options that may be considered?  Have there been situations in which you considered conservative kidney management but ultimately did not mention it to the patient? | Could you describe this in more detail?  Why was that? |
|  | - Assessing individual risks and prognosis / life expectancy | How do you experience estimating individual disease trajectories, including the risks of each treatment option, in order to provide recommendations? | To what extent?  Why? |
|  | - Availability of tools/materials (e.g., survival prognosis tools, patient decision aids) | What options do you have in your daily work to determine individual risks and survival prognoses? | How do you experience this?  How effective are these options in supporting your decision-making?  To what extent does this help you in the decision-making process together with patients?  What would help you to be able to provide such information? |
|  | - Assessing patients’ needs and preferences | How do you proceed in order to explore patients’ needs and preferences?  How confident/structured do you feel in assessing needs and preferences? | Could you explain this in more detail? |
|  | - Perception of conservative kidney management by patients and relatives | Based on your experience, how is the concept of conservative kidney management perceived by patients and/or relatives? | How does this influence whether you continue to consider this treatment option? |
|  | - Addressing end-of-life / dying for the first time | How confident do you feel in end-of-life discussions? | What would help you to feel more confident? |
| *Topic 2: Delivery of conservative kidney management* | | | |
| How feasible is it for you to follow the pathway of CKM in your daily routine? | - Interdisciplinary collaboration | How relevant is the collaboration with other specialties/disciplines for implementing conservative kidney management to you?  Which specialties/disciplines do you collaborate with in the context of conservative kidney management?  To what extent is collaboration with other specialties/disciplines possible in the context of conservative kidney management?  How are responsibilities shared in interdisciplinary collaboration? | Could you describe this in more detail? |
|  | - Familiarity with processes/guidelines | How confident do you feel in providing the conservative care pathway (e.g., symptom and medication management, crisis plans)? | What would you need in order to feel more confident? |
|  | - Time capacities | How well do your available time resources fit with providing conservative kidney management? | Could you describe this in more detail? |
| *Topic 3: Perception of conservative kidney management* | | | |
| What beliefs or opinions do you have about CKM? | - Conservative kidney management vs. physician identity (“passive approach”) | To what extent does conservative kidney management align with your professional role identity as a nephrologist? | Could you describe this in more detail?  To what extent does this influence whether conservative kidney management is mentioned as a treatment option? |
|  | - Feeling of “giving up” on the patient | To what extent do you feel that you are actively treating patients when conducting conservative kidney management? | Could you describe this in more detail?  How do you feel about it? |
| How strongly does it influence your relationship with patients if you express doubts about dialysis as a treatment option? | - Disappointing patients (expectation: “the doctor will help me”) | To what extent do you feel you are meeting patients’ expectations in this context? | Why ist hat?  What influence does this have on your decision to provide this care pathway? |
|  | - Losing (long-term) patients | To what extent is there a fear of losing patients because of expressing doubts about dialysis? | Could you explain that a bit more? |
| *Topic 4: Barriers and facilitators in implementing CKM* | | | |
| What do you think are the barriers and facilitators to implementing CKM (in your daily routine)? | - Financial aspects (reimbursement system) | What role does reimbursement play in choosing between dialysis treatment and conservative kidney management? | Could you specify this? |
|  | - Influence of colleagues | What role do the attitudes or views of colleagues (e.g. other nephrologists), general practitioners, or nurses/medical assistants play in your decision to provide conservative kidney management? | Could you describe this in more detail? |
|  | - Training (e.g., communication training, ...) | What training needs do you see, and which topics would you find most useful? |  |
| *Closing* | | | |
| We have now reached the end of the interview.  Is there anything you would like to add that has not yet been discussed regarding your experiences or views on conservative kidney management? |  |  |  |

## [Stop audio recording]

Closing Remarks

Thank you very much for your participation.

Can you recommend another nephrologist who works with this patient group that I can contact?

For the description of the sample, I would like to collect some additional data. I would therefore ask you to complete the following table [handing out table eliciting participant information].
