# Supplementary material for: Barriers and facilitators of conservative kidney management in older adults with chronic kidney failure: a qualitative study with nephrologists in Germany
Source: BMC Nephrol. 2026 Oct 31;27:646. doi: 10.1186/s12882-026-05470-3 (PMC13732868; doi:10.1186/s12882-026-05470-3)
Supplement: Supplementary file 2 [file 12882_2026_5470_MOESM2_ESM.docx]

**Additional file 1: Research team and reflexivity**

| **Topic** | **Description** |
| --- | --- |
| Credentials | JK and MHB hold a master degree and MHB is currently a PhD candidate. ES, NE, CMK and NM hold doctoral degrees, with ES and NE holding medical doctorates. ES also holds a professorship in health services research and nephrology. |
| Occupation | At the time of the study, JK was a master’s student in health services research and worked as a study assistant at the Institute of Public Health, Charité - Universitätsmedizin Berlin (IPH). MHB, ES, NM and NE worked as research associates at the IPH, with ES serving as the Institute´s Deputy Director. CMK worked as research associate at the Institute for Health Services and Health System Research, Brandenburg Medical School (Theodor Fontane). |
| Gender | JK, ES, NM, CMK and NE are female, MHB is male. |
| Experience and Training | JK, CMK and MHB have experience in qualitative research, including conducting and analyzing interviews. ES and NE are nephrologists. ES is also an epidemiologist, as in NM, who has a background in molecular biology. MHB, CMK, ES, NM and NE have published in both quantitative and qualitative research. |
| Relationship established | No prior relationship was established between the interviewer and the interviewees. |
| Participant knowledge of the interviewer | Interviewees were aware of the interviewer’s background and that the study was being conducted as part of her master thesis. |
| Interviewer characteristics | The interviewer was a master´s student in health services research. She has a background in nursing and speech language therapy. She has experience in caring for patients with kidney diseases excluding dialysis care. JK had prior knowledge of potential barriers to CKM, which may have influenced her assumptions and interpretation of the data. To include different perspectives, coding decisions and interpretations were discussed with MHB. The research question was developed in collaboration with the research team at the IPH. The interviewees were informed that the interviewer was not a physician. This may have encouraged the nephrologists to speak more openly about barriers, as they may have felt less concerned about being professionally judged. |
